# Supplementary material for: Highly efficient A-to-G base editing by ABE8.17 in rabbits
Source: Mol Ther Nucleic Acids. 2022 Jan 28;27:1156–63. doi: 10.1016/j.omtn.2022.01.019 (PMC8888895; doi:10.1016/j.omtn.2022.01.019)
Supplement: Document S1. Figures S1–S4 and Tables S1–S5 [file mmc1.pdf]

OMTN, Volume *27*

Supplemental information

Highly efficient A-to-G base editing by ABE8.17 in rabbits

Ding Zhao, Yuqiang Qian, Jinze Li, Zhanjun Li, and Liangxue Lai

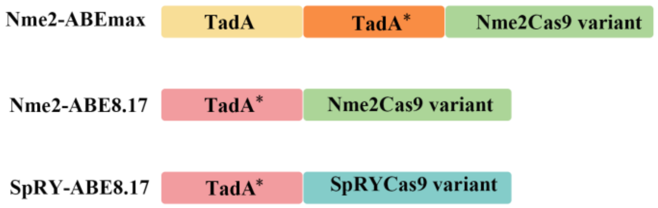

Supplementary Figure 1

The architecture of Nme2-ABEmax, Nme2-ABE8.17 and SpRY-ABE8.17.

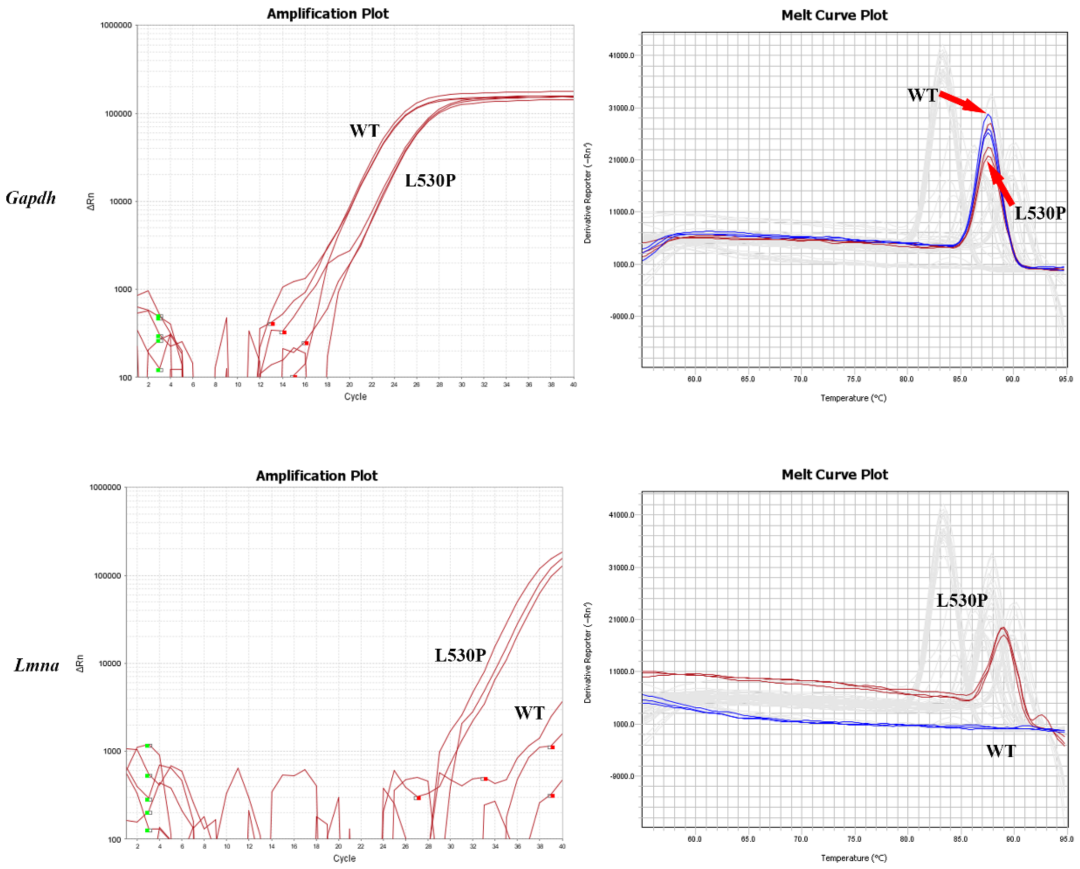

Supplementary Figure 2

Expression of the *Lmna* gene was determined by qRT–PCR with specific primers targeting the exon and intron.

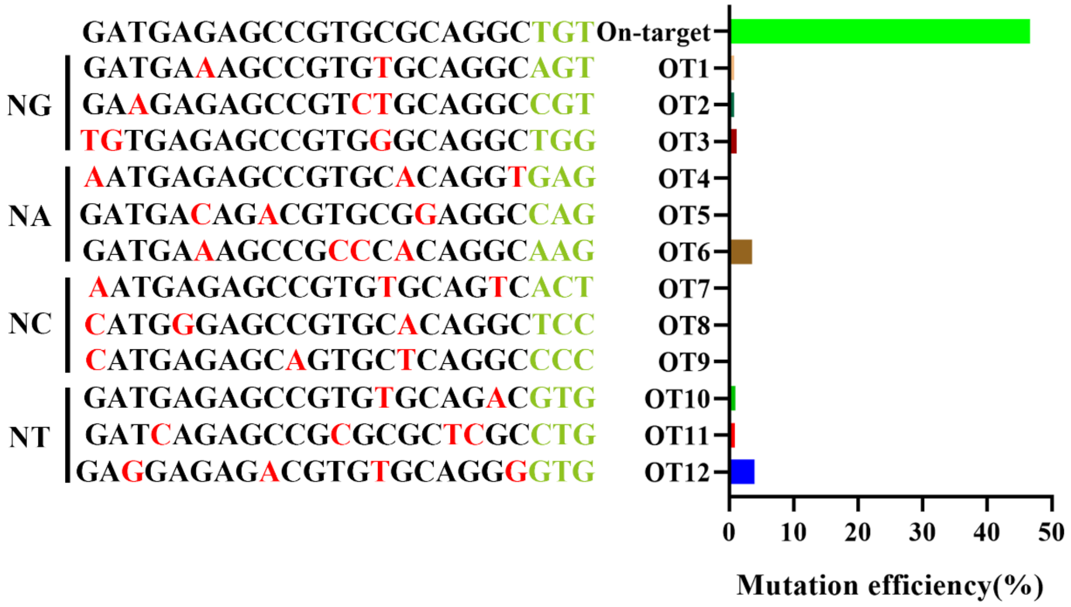

Supplementary Figure 3

Analysis of off-target effects in *Lmna* mutant rabbits by deep sequencing of HiTOM analysis. The potential off-target sites (POTs) in the rabbit genome for sgRNA were predicted to analyze site-specific edits with Cas-OFFinder (http://www.rgenome.net/cas-offinder/). Deep sequencing was carried out to analyse the PCR products of the POTs. All the primer sequences for the off-target assay are listed in Data S1.

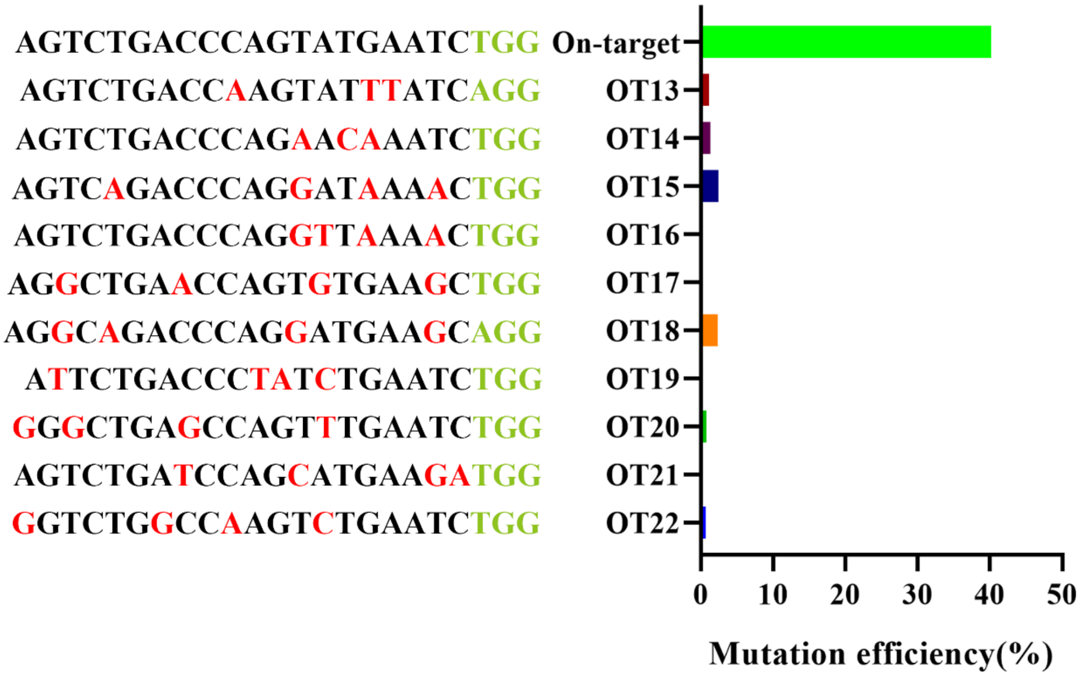

Supplementary Figure 4

Analysis of off-target effects in *Tyr* mutant rabbits by deep sequencing of HiTOM analysis. The potential off-target sites (POTs) in the rabbit genome for sgRNA were predicted to analyze site-specific edits with Cas-OFFinder (http://www.rgenome.net/cas-offinder/). Deep sequencing was carried out to analyze the PCR products of the POTs. All the primer sequences for the off-target assay are listed in Data S1.

Table S1 Summary of mouse embryo base editing rate using ABE8.17 system

Target gene	Base editor	No. of blastocysts	Mutant ratio (%)	
			No. of mutants	No. of target mutants
Tyr	ABE7.10	7	0(0)	0(0)
	ABE8.17	13	12(92)	12(92)
Dmd	ABE7.10	9	0(0)	0(0)
	ABE8.17	11	4(36)	3(27)
Lmna	ABE7.10	11	5(45)	0(0)
	ABE8.17	9	9(100)	3(33)

Table S2 Summary of rabbit embryo base editing rate using ABE8.17 system

Target gene	Base editor	No. of blastocysts	Mutant ratio (%)	
			No. of mutants	No. of target mutants
Tyr-1	ABE7.10	6	0(0)	0(0)
	ABE8.17	5	4(80)	4(80)
Tyr-2	ABE7.10	7	0(0)	0(0)
	ABE8.17	9	3(33)	3(33)
Lmna	ABE7.10	9	3(33)	0(0)
	ABE8.17	11	9(82)	4(36)
	SpRY-ABE8.17	9	5(56)	5(56)

Table S3. Target sites in human cells used in this study. Target nucleotides (red)

Target site	sequence (5'-3')	PAM
**ABE-site1**	GAAGA_5CCA_8AGGATAGACTGC	GGG
**ABE-site2**	GAACA_5CA_7AAGCATAGACTGC	GGG
**ABE-site3**	GATGA_5GA_7TA_9ATGATGAGTCA	AGG
**ABE-site4**	GATGA_5G$A_7$$A_8$GGAGAAGTTCTT	TGG
**ABE-site5**	GAATA_5CTA_8AGCATAGACTCC	AGG
**ABE-site6**	GTGT$A_5$$A_6$GACCTCAAAAGCAC	AGG
**ABE-site7**	AAAT$A_5$$A_6$TGCCATCTTCCGCT	AGG
**NAA**	GGCTGCACAACCAGTGGAGG	CAA
**NAC**	GGGTCAGACGTCCAAAACCA	GAC
**NAT**	GTCCACTTCCCCTCCCACTA	CAT
**NAG**	GCAGGAGGTGGGGAAGGCCG	AAG
**NGA**	GAGGAGGAAGGGCCTGAGTC	CGA
**NGC**	GACA_4GGCA_8GGGGCACCGCGG	CGC
**NGG**	AAAT$A_5$$A_6$TGCCATCTTCCGCT	AGG
**NGT**	GTTCCAGAACCGGAGGACAA	AGT
**Nm1**	ATCTGTCCCCTCCACCCCACAGT	GGGGCC
**Nm2**	ATGTTCCAGCTTCCTGGGTCTGC	AGGTCC

Table S4. Primers used for genotyping in this study

**Target site**	**Sequence (5'-3')**
**ABE-site1**	F GAGGCATGAGGATCACTTGAG
	R TCAGCTCCAACAGCACTTT
**ABE-site2**	F ACTGCCATTCTACCAACAATAGA
	R AAGCAGGTGATTACAGGATTGA
**ABE-site3**	F ACAGTGGGTTAATAGGAGAATGAG
	R TCCCAAAGTGCTGGGATTT
**ABE-site4**	F CACCCCTTCAGTCCATGCTT
	R TCTGATGGGGAGGAACGAGT
**ABE-site5**	F AGAAGGTAAGTGCATGGTAAGG
	R CCAGATTTCTCAGCCTCTTTCT
**ABE-site6**	F GGAGAACCATAGGCAGAAGAAT
	R CTACTGAGTTGCTTGAGCTCTTA
**ABE-site7**	F GCGAGGGTTACAGTTCTGTTTAG
	R GCCTCCTGCTGTCATTGATATT
**NAA**	F CATTTCGCGGATGTTCCAATC
	R CCTGGAGATTTGGGTTCTCTC
**NAC**	F CTCTGGTGCTGTGTGACTA
	R GGACGAGGGAAATTTGAAATCG
**NAT**	F AAGCGATGAGCAAGCTACC
	R AGAACCTCATTCAGTGGTTCATTA
**NAG**	F CGTTACCACCTGGTGCAG
	R CCCAGAAGCCAGTGGAC
**NGA**	F TAGCCTCAGTCTTCCCATCA
	R GTTGCCCACCCTAGTCATT
**NGC**	F GACAGACCACAAGCAGGTAAA
	R TTTCAACCCGAACGGAGAC
**NGG**	F GCGAGGGTTACAGTTCTGTTTAG
	R GCCTCCTGCTGTCATTGATATT
**NGT**	F GGGGCCTCCTGAGTTTCTCATCTG
	R GGTTGCCCACCCTAGTCATTGGAG
**Nm1**	F CAGCTAGTCTTCTTCCTCCAAC
	R CCAGATAAGGAATCTGCCTAACA
**Nm2**	F TCTTCTCCTGTGGTGGAGT
	R CTGTGCCTCAACCTCCTAAC
Tyr	F TGCCAGAAAGCTGAATGATACT
	R CTTCGCTTCCCTTTCCCTTT
Dmd	F TGGAGTTATATCCCTGTGGAAAC
	R CCAAGATAGGCCTCATGACAA
***Lmna* (mouse)**	F CCACCGAAGTTCACCCTAAA
	R GGCTGACTTCCACCCATTAT
Tyr-1	F GTACAATAGCCGACAGAGCTTAT

	R CTGCCTGGTGTTTCTGTTTATTT
Tyr-2	F CTCCCAGTGATGAGAAACCTAAC
	R CTCCCAGTGATGAGAAACCTAAC
***Lmna* (rabbit)**	F GACAGTAGCACGGAGTGAG
	R GCTAAGGAACTTGCTCCCA
**PolyA-1**	F CCCAATTCCACGATGAACAGA
	R CTCCAAAGCTGAAGGCAAGA
**PolyA-2**	F AGTTGTTACCGTTGGGACTATTT
	R AAGATCGCGCCATTGTACTC
**PolyA-3**	F GGGCATTTGGAAGTCAGTAGTA
	R TTTGGGAGGTGATCAGGTTATG
**PolyA-4**	F CGACAACACTTGTTATGGAAAGG
	R AGACTGGGAAAGGCAATGG
**PolyA-5**	F GTATGTGAGTGACCACCAAGAG
	R CTCCCTCTTATCCATGTGTTAGC
**PolyA-6**	F ACCTGTAATCCTAGCACTTTGG
	R AGCAAGGAGCAACAGAAGAA
Atm	F TTTGTATGGCTGTGGTGGAG
	R TTTCCTGTGTCTCCCTGAATTT
Mtm1	F GGATTATTGATTTGGCACCCTTATT
	R TCTCCAGTCTGGTATGGTACTT
Serpina1	F AGCTCCTTGACCAAATCCAC
	R CCAGTCCAACAGCACCAATA
Vps13b	F GCTTTCTGCCATAATGAAGTCTG
	R AAAGAGGGCTGAACACAATCTA
Th	F CTTGTCCAGCTCTGACACTT
	R TCTCCTTCTCTCTCTCTTCCTG
Scnn1g	F CCTCACTGTGGCTGGTTTAT
	R GGGAGAATCTAGGCTGCTTTC
Lipa	F ACAACTTCAGAGTTACCACCTATC
	R TACCTTGCCAGTGCTGTTT

Table S5. Generation of *Lmna* and *Tyr* rabbits using ABE8.17 systems.

Target site	Embryos transferred	No. of offspring	No. of mutants	No. of target mutants	Frequencies of target mutations
Lmna (p.L530P)	42	7	2(28.57%)	1(14.29%)	45.41%
Tyr (p.T325A)	46	5	3(60.00%)	3(60.00%)	(40.03±26.82%)

Data are presented as mean ± SD.
