# Supplementary material for: Highly efficient A-to-G base editing by ABE8.17 in rabbits
Source: Mol Ther Nucleic Acids. 2022 Jan 28;27:1156–63. doi: 10.1016/j.omtn.2022.01.019 (PMC8888895; doi:10.1016/j.omtn.2022.01.019)
Supplement: Document S2. Article plus supplemental information [file mmc3.pdf]

Molecular Therapy
Nucleic Acids
Original Article

Highly efficient A-to-G base editing by ABE8.17 in rabbits

Ding Zhao,[1,5] Yuqiang Qian,[1,5] Jinze Li,[1,5] Zhanjun Li,[1] and Liangxue Lai[1,2,3,4]

[1]Key Laboratory of Zoonosis Research, Ministry of Education, Jilin University, Changchun 130062, China; [2]CAS Key Laboratory of Regenerative Biology, Guangdong Provincial Key Laboratory of Stem Cell and Regenerative Medicine, South China Institute for Stem Cell Biology and Regenerative Medicine, Guangzhou Institutes of Biomedicine and Health, Chinese Academy of Sciences, Guangzhou 510530, China; [3]Guangzhou Regenerative Medicine and Health Guang Dong Laboratory (GRMH-GDL), Guangzhou 510005, China; [4]Institute for Stem Cell and Regeneration, Chinese Academy of Sciences, Beijing 100101, China

Adenine base editors (ABEs), composed of an evolved adenine deaminase fused to the Cas9 nickase, enable efficient and precise A-to-G conversion in various organisms. However, the base editing of some challenging loci with the ABE7.10 system in rabbits was inefficient in our previous study. Here, we show that ABE8.17 and SpRY-ABE8.17 can efficiently induce base editing in mouse and rabbit embryos. In addition, this strategy can be used to precisely mimic clinical point mutations in rabbits. Furthermore, by eliminating the linker in ABE8.17, we created ABE8.17-NL, which achieved efficient base editing within a narrowed window (2–4 nts) in human HEK293FT cells. Collectively, these findings show that ABE8.17 systems can efficiently induce efficient A-to-G base editing at desired sites and that the ABE7.10 system is inefficient, thus providing an efficient way to generate ideal disease models in rabbits.

INTRODUCTION

Adenine base editors (ABEs), containing an evolved *Escherichia coli* tRNA-specific adenosine deaminase (TadA) and a catalytically impaired Cas9 protein (nCas9), can efficiently convert A to G with little accompanying double-stranded DNA breaks (DSB) generation.[1] The ABE7.10 system has been applied to efficiently introduce single nucleotide modifications in various plants[2,3] and animals.[4–8] Recently, evolved TadA8 variants (TadA-8e and TadA-8s) were developed by introducing additional mutations into TadA-7.10.[9,10] Compared with TadA-7.10 monomers, TadA-8e and TadA-8s monomers support more efficient A-to-G base conversion with SpCas9 (ABE8e and ABE8s). Our previous study demonstrated that the ABE7.10 system provides a simple and highly efficient method for inducing single-nucleotide substitutions in rabbits.[8] However, some challenging loci were unable to be effectively base edited by ABE7.10 in the previous study.[8]

To efficiently edit these challenging loci, ABE8 systems were used in this study. Our results show that ABE8.17 enables efficient A-to-G base editing at desired loci in rabbits. In addition, we found that ABE8.17 can edit multiple A bases in a broad window within the protospacer in rabbit embryos. Thus, we created ABE8.17-NL (no linker) by deleting the linker to narrow the window of activity, improving the precision of ABE8.17.

Overall, our findings demonstrate that ABE8.17 provides a highly efficient system for the desired single-nucleotide substitutions in rabbits. By eliminating the linker sequence, we generated ABE8.17-NL, an optimized ABE, narrowing the editing window to 2–4 nts.

RESULTS

Highly efficient A-to-G base conversion using ABE8s in human cells

We first assessed the activities of ABE7.10, ABEmax, ABE8.17, ABE8.20, and ABE8e in human HEK293FT cells (Table S3). ABEmax and ABE7.10 consist of the N-terminal wild-type (WT) TadA monomer fused to a C-terminal-evolved TadA monomer,[1,11] while ABE8.17, ABE8.20, and ABE8e consist of an evolved TadA monomer fused to a D10A nickase Cas9 domain.[9,10] (Figure 1A) Sanger sequencing results revealed a significantly increased base editing-efficiency when using the ABE8.17, ABE8.20, and ABE8e variants relative with the ABEmax and ABE7.10 variants (Figure 1B). In addition, the editing efficiency of ABE8.17 at 4 of 7 tested loci was slightly higher than that of with ABE8e and ABE8.20 (Figure 1B). Thus, ABE8.17 was used in the following study.

To expand the targeting scope of ABE8.17, we investigated whether SpRYCas9[12] (a Cas9 variant, PAM: NRN > NYN) could enable efficient A-to-G conversion in human HEK293FT cells by fusing it to TadA-8.17 (Figure S1). As shown in Figure 1C, efficient base editing (3%–48%) was achieved at 8 sites by using SpRY-ABE8.17 (Table S3). In addition, a significant increase in base editing was achieved by using Nme2-ABE8.17 relative to Nme2-ABEmax, which was shown to be an inefficient base-editing system in our previous study (Figures 1D, and S1; Table S3).[13]

Received 28 April 2021; accepted 24 January 2022;
https://doi.org/10.1016/j.omtn.2022.01.019.

[5]These authors contributed equally

Correspondence: Zhanjun Li, PhD, Key Laboratory of Zoonosis Research, Ministry of Education, Jilin University, Changchun 130062, China.
E-mail: lizj_1998@jlu.edu.cn
Correspondence: Liangxue Lai, PhD, Key Laboratory of Zoonosis Research, Ministry of Education, Jilin University, Changchun 130062, China.
E-mail: lai_liangxue@gibh.ac.cn

 © 2022 The Author(s).
This is an open access article under the CC BY license (http://creativecommons.org/licenses/by/4.0/).

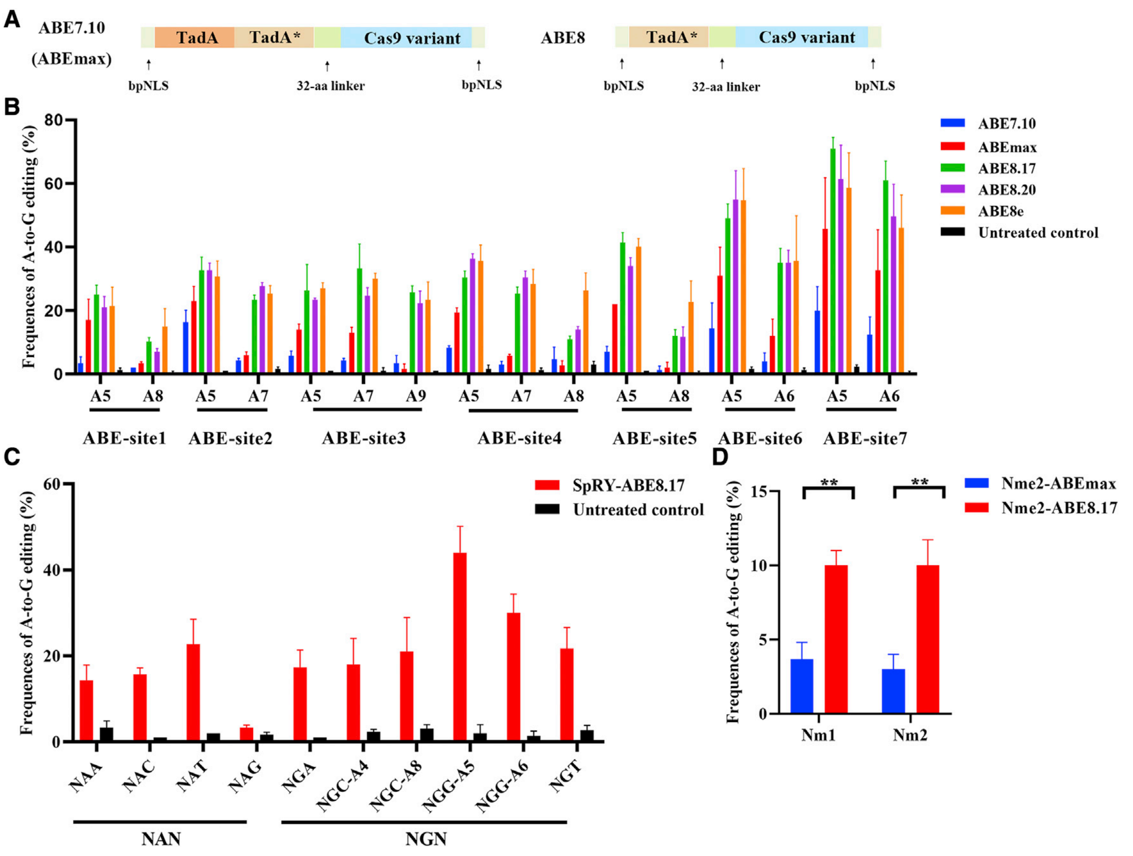

Figure 1. Highly efficient A-to-G base conversion using ABE8 in human cells

(A) The architecture of ABE7.10, ABEmax, and ABE8. NLS, bipartite nuclear localization signal. (B) Base-editing efficiency of ABE7.10 versus ABEmax, ABE8.17, ABE8.20, and ABE8e in HEK293FT cells. Bars represent mean values, and error bars represent the SD of three independent biological replicates. (C) Base-editing efficiency at eight target sites harboring NRN PAMs in HEK293FT cells by SpRY-ABE8.17. Bars represent mean values, and error bars represent the SD of three independent biological replicates. (D) Base-editing efficiency of Nme2-ABEmax versus Nme2-ABE8.17 in HEK293FT cells. Bars represent mean values, and error bars represent the SD of three independent biological replicates. For editing across the entire protospacer for each site, see Table S3. $^{**}p < 0.01$, two-tailed unpaired t-test.

Highly efficient A-to-G base editing by ABE8.17 in mouse and rabbit embryos

First, 3 target loci (*Tyr*, *Dmd* and *Lmna*) were used to test the efficiency of ABE8.17 in mouse embryos (Figure 2A). Base editing was conducted in mouse embryos via the microinjection of ABE8.17-encoding mRNA or ABE7.10-encoding mRNA, and single-guide RNAs (sgRNAs). Sanger sequencing results showed that 12 of the 13 desired edits in *Tyr* exhibited efficiencies ranging from 10% to 80% (Figure 2B; Table S1). Four of the 11 desired edits in *Dmd* showed efficiencies ranging from 8% to 79% when using ABE8.17 but no desired base-editing activity at these two loci when using ABE7.10 (Figure 2C; Table S1). A significantly increased base-editing efficiency was also achieved at the A6 and A9 sites of the *Lmna* gene by ABE8.17 relative to ABE7.10 (Figure 2D; Table S1).

Then ABE8.17 was used to target the base editing of 3 rabbit loci, which was inefficient when using the ABE7.10 system in our previous study[8] (Figure 2E). Sanger sequencing results showed that 3 of the 9 desired edits in *Tyr-1* presented efficiencies ranging from 10% to 18% and that 4 of the 5 desired edits in *Tyr-2* presented efficiencies ranging from 41% to 72% when using ABE8.17 (Figure 2F and 2G; Table S2). However, none of the desired mutations in these two loci were detected when using ABE7.10 (Figures 2F and 2G; Table S2). Similar results were obtained for the *Lmna* gene (Figure 2H and Table S2), suggesting that ABE8.17 was more efficient than ABE7.10. Then, the SpRY-ABE8.17 with a relaxed PAM was used to target the desired A5 mutations at the *Lmna* locus. Sanger sequencing results showed that 5 of the 9 desired edits of A5 were achieved using SpRY-ABE8.17 with efficiencies ranging from 22% to 40% (Figure 2I; Table S2).

These results indicated that the ABE8.17 and SpRY-ABE8.17 systems were efficient at the desired loci than ABE7.10 in mouse and rabbit embryos, suggesting the potential use of ABE8.17 to develop animal models for human genetic diseases in rabbits.

ABE8.17 can induce efficient A-to-G conversion in rabbits

The majority of known human genetic diseases are caused by point mutations, with C-to-T or A-to-G mutations accounting for

A

Target gene	Phenotype	Sequence
Tyr	WT	AGTTTGACCCAGTATGAATCTGG
(p.T325A)	OCA1	AGTTTGGCCCAGTATGAATCTGG
Dmd	WT	GATCACAGTCAGTCTAGCACAGG
(p.T272A)	DMD	GATCGCGGTCAGTCTAGCACAGG
Lmna	WT	CCTTCGCACCGCTCTCATCAACT
(p.L530P)	EDMD	CCTTCGCACCGCTCCCATCAACT

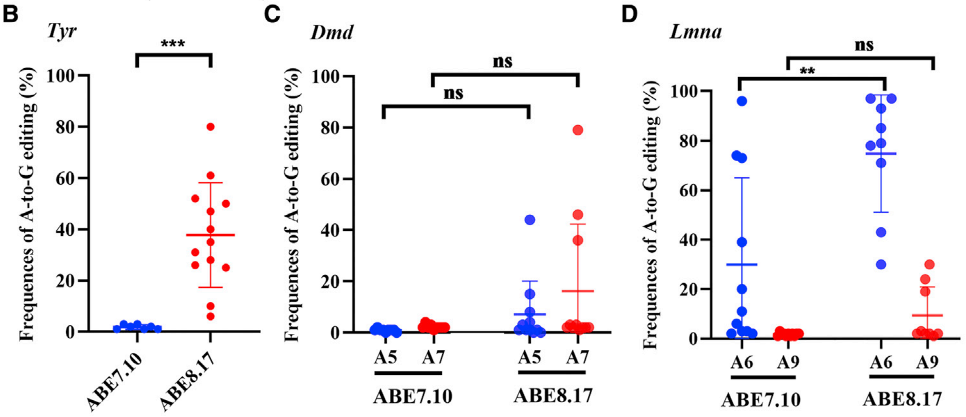

E

Target gene	Base editor	Phenotype	Sequence
Tyr-1	ABE7.10/ABE8.17	WT	TTTCAGCTTTAGAAATACACTGG
(p.S339G)		OCA1	TTTCGGCTTTAGAAATACACTGG
Tyr-2	ABE7.10/ABE8.17	WT	AGTCTGACCCAGTATGAATCTGG
(p.T325A)		OCA1	AGTCTGGCCCAGTATGAATCTGG
Lmna	ABE7.10/ABE8.17	WT	CCTGCGCACGGCTCTCATCAATT
(p.L530P)		EDMD	CCTGCGCACGGCTCCCATCAATT
	SpRY-ABE8.17	WT	ACAGCCTGCGCACGGCTCTCATC
		EDMD	ACAGCCTGCGCACGGCTCCCATC

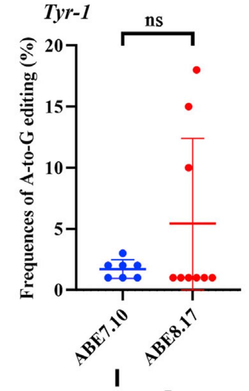

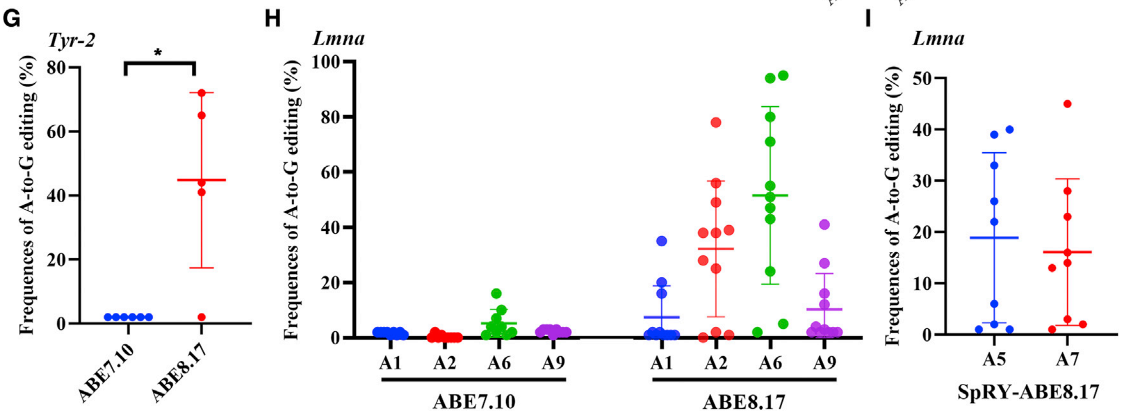

Figure 2. Highly efficient A-to-G base editing by ABE8.17 in mouse and rabbit embryos

(A) Target-site sequences within the targeted loci in mouse embryos. Target sequence (black), PAM region (green), target sites (red), and mutant amino acid (underlined). WT, wild type. (B–D) The A-to-G editing frequencies at the target site using ABE8.17 and ABE7.10. A5 and A7 indicates the edited positions in the protospacer for *Dmd*; A6 and A9

(legend continued on next page)

approximately half of all known pathogenic SNPs.[1] Notably, rabbits are considered better animal models than mice in recapitulating some human diseases because of the higher similarity of their physiology, anatomy and genetics to those of humans.[14]

Here, two mutation sites in *Tyr* (p.T325A) and *Lmna* (p.L530P) were used for A-G base editing by ABE8.17 in rabbits. The *Tyr* mutation (p.T325A) is the major causal genetic mutation responsible for human ocular albinism (OA) and oculocutaneous albinism (OCA).[15,16] OCA1 is an autosomal recessive disorder characterized by reduced or absent melanin pigment in the skin, hair, and eyes due to deficient tyrosinase catalytic activity.[17] As shown in Figures 3A and 3B; Table S5, 3 of the 5 desired *Tyr* (p.T325A) mutations were identified in rabbit pups by using Sanger sequencing, and the results of targeted deep sequencing showed 66.72%, 13.09%, and 40.27% *Tyr* mutation efficiencies in pups #1, #2, and #5, respectively (Figure 3C).

A point mutation (p.L530P) in the *Lmna* gene results in the development of Emery-Dreifuss muscular dystrophy (EDMD), which is characterized by early elbow and Achilles tendon contracture, slow and progressive muscle atrophy and weakness, and cardiomyopathy with conduction block.[18] As shown in Figures 3D and 3E; Table S5, 1 of the 7 desired *Lmna* mutations (p.L530P) mutations was identified in rabbit pups (#6) by using Sanger sequencing, and the results of targeted deep sequencing showed a 45.41% desired *Lmna* mutation efficiency in rabbit pup #6 (Figure 3F). In addition, decreased in lamin A/C protein expression relative to the WT rabbits was identified in rabbit pup #6 (p.L530P) by western blotting (Figure 3G). In addition, the retention of 21 bp of intron 9 of *Lmna* was observed by using quantitative real-time polymerase chain reaction (PCR) with specific primers targeting the exon and intron (Figures 3H, 3I, and S2), which was also confirmed by Sanger sequencing (Figure 3J).

Furthermore, no undesirable base change or sgRNA sequence-dependent off-target mutations were observed by deep sequencing in these rabbits, indicating the efficiency and precision of ABE8.17 in rabbits (Figures S3 and S4; Data S1). These results show that ABE8.17 and SpRY-ABE8.17 can induce efficient A-to-G conversion and show great promise as base editing tools for the generation of rabbit disease models.

Efficient base editing with a narrow window using ABE8.17-NL

Although ABE8.17 can induce efficient A-to-G conversion, the large window within the protospacer is a substantial limitation when multiple A bases are located in the sgRNA. Here, the ABE8.17-NL (no linker) system was used to narrow the editing window in HEK293FT cells (Figure 4A).

We initially investigated the effects of base-editing precision and efficiency when six polyA sites were targeted by using the deleted linker sequence between TadA-8.17 and nCas9. As shown in Figures 4B–4G, the base-editing window was significantly narrowed by using ABE8.17-NL relative to ABE8.17, which contained the $(SGGS)_2$-XTEN-$(SGGS)_2$ linker (32 amino acids initially included in the ABE7.10 construct to improve editing efficiency).[1] Then, we tested the effects of several sites containing A bases within the window on the base editing precision and efficiency by using ABE8.17-NL (Figure 4H). The results showed that the window from A3 to A6 (4 nts) was narrowed by using ABE8.17-NL relative to the large editing window from A3 to A8 (6 nts) and that high activity at A4 and A5 was maintained using ABE8.17(Figure 4I). Taken together, these results demonstrate that ABE8.17-NL can induce efficient base editing within a narrow window (2–4 nts) in human HEK293FT cells.

DISCUSSION

In this study, we confirmed that the ABE8.17 system enables robust A-to-G base editing at loci that are difficult to edit by using ABE7.10 in rabbits. First, we demonstrated that the ABE8 variants showed robust A-to-G base-editing in HEK293FT cells. Second, TadA8.17 was demonstrated to be compatible with SpRYCas9, providing an opportunity to eliminate the constraints imposed by PAM availability. Furthermore, this strategy can be used to precisely mimic human pathologies by efficiently inducing point mutations in rabbits. These results demonstrated the high efficiency of A-to-G base editing by ABE8.17 in rabbits.

There was no significant off-target effect of predicted gRNA-dependent sites in the base-editing rabbits in this study. The gRNA-independent sites also need to be checked. However, previous studies demonstrated that base editors may cause genome-wide off-target effects on DNA and RNA,[19,20] and sgRNA-independent off-target effects are mainly induced by the deaminase domain rather than the Cas9 domain. In addition, there are many gaps in the published rabbit genome, which is a major limitation of whole-genome sequencing. Thus, we focused on the sgRNA dependent off-target effects in this study.Deep sequencing results demonstrated that ABE8.17 and SpRY-ABE8.17 could induce site-specific, single-base substitutions with efficiency rates of 13.09–66.72% in F0 rabbit pups, which is induced by mosaicism mutations after the microinjection of the embryos, causing unequal genome editing in individual blastomeres.[21] These results confirmed that the mosaicism generated by CRISPR/Cas9 was commonly detected in F0 gene-edited pups.[8,22,23]

High-precision base editor systems represent essential tools for the future application of DNA editing, especially in gene therapy. However, ABE8.17 showed an expanded window within the protospacer when multiple A bases were located in the sgRNA, which is also a major limitation of the future application of ABE8.17. Therefore, we generated ABE8.17-NL, which reduced the width of the editing window

indicate the edited positions in the protospacer for *Lmna*. *** $p < 0.001$, two-tailed unpaired t-test or unpaired t-test with Welch's correction. (E) Target-site sequences within the targeted loci in rabbit embryos. Target sequence (black), PAM region (green), target sites (red), and mutant amino acid (underlined). WT, wild type. (F–H) The A-to-G editing frequencies at the target site using ABE8.17 and ABE7.10. A1, A2, A6, A9, A5, and A7 indicate edited positions in the protospacer for *Lmna*. ns, no significance. *$p < 0.05$, **$p < 0.01$, ***$p < 0.001$, unpaired t-test with Welch's correction. (I) The A-to-G editing frequencies achieved in *Lmna* (p.L530P) using SpRY-ABE8.17.

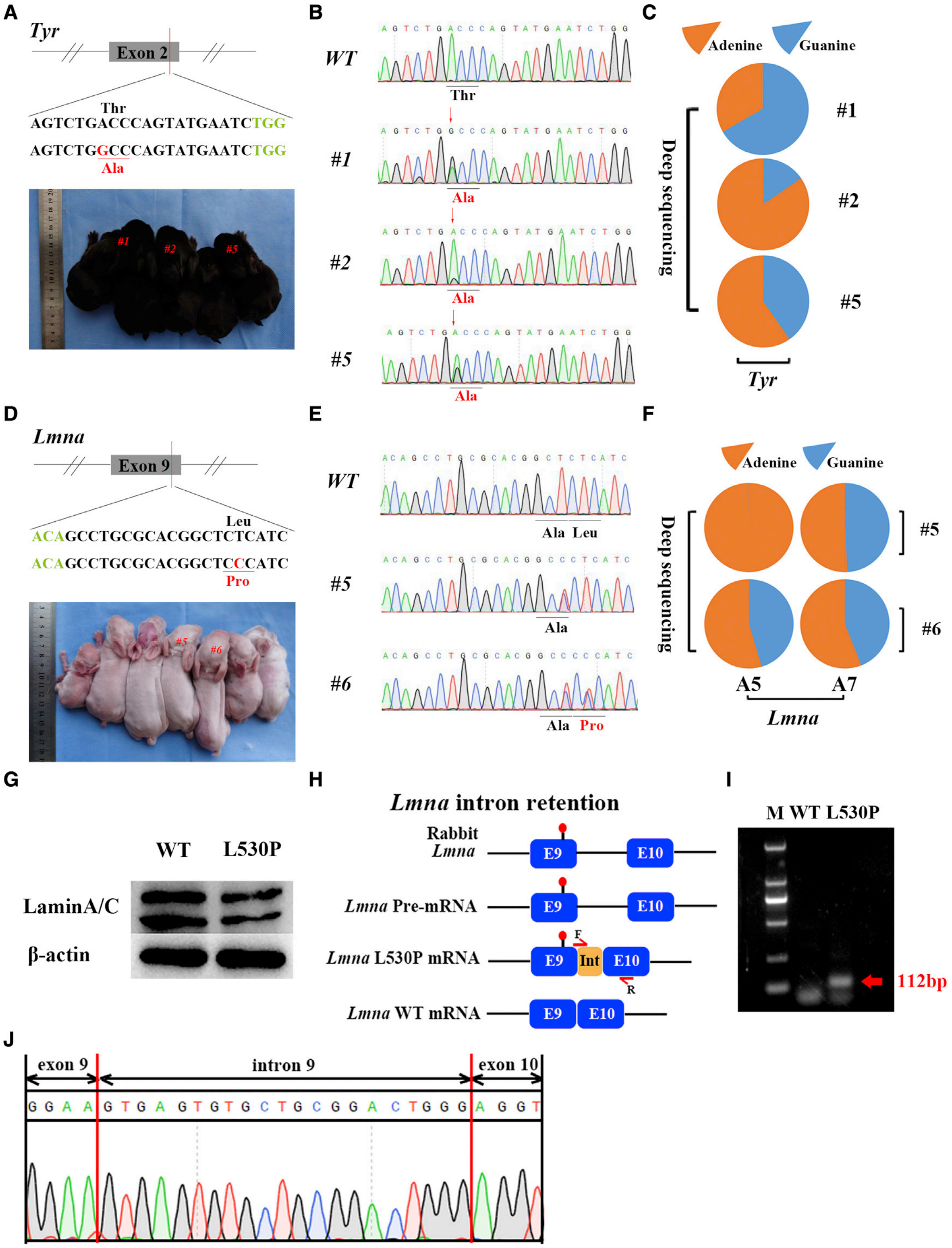

Figure 3. ABE8.17 and SpRY-ABE8.17 can induce efficient A-to-G conversion in rabbits

(A) The target sequence at the *Tyr* (p.T325A) locus. The PAM- and sgRNA-targeted sequences are shown in green and black, respectively. The substituted bases are indicated in red. (B) Sanger sequencing chromatograms of DNA from WT and *Tyr* (p.T325A) rabbits (#1, #2, and #5). The red arrow indicates the substituted nucleotide. Relevant codon identities at the target site are presented beneath the DNA sequence. (C) Characterization of the targeted modifications in *Tyr* (p.T325A) rabbits by deep sequencing. (D) The target sequence at the *Lmna* (p.L530P) locus. The PAM- and sgRNA-targeted sequences are shown in green and black, respectively. The substituted

(legend continued on next page)

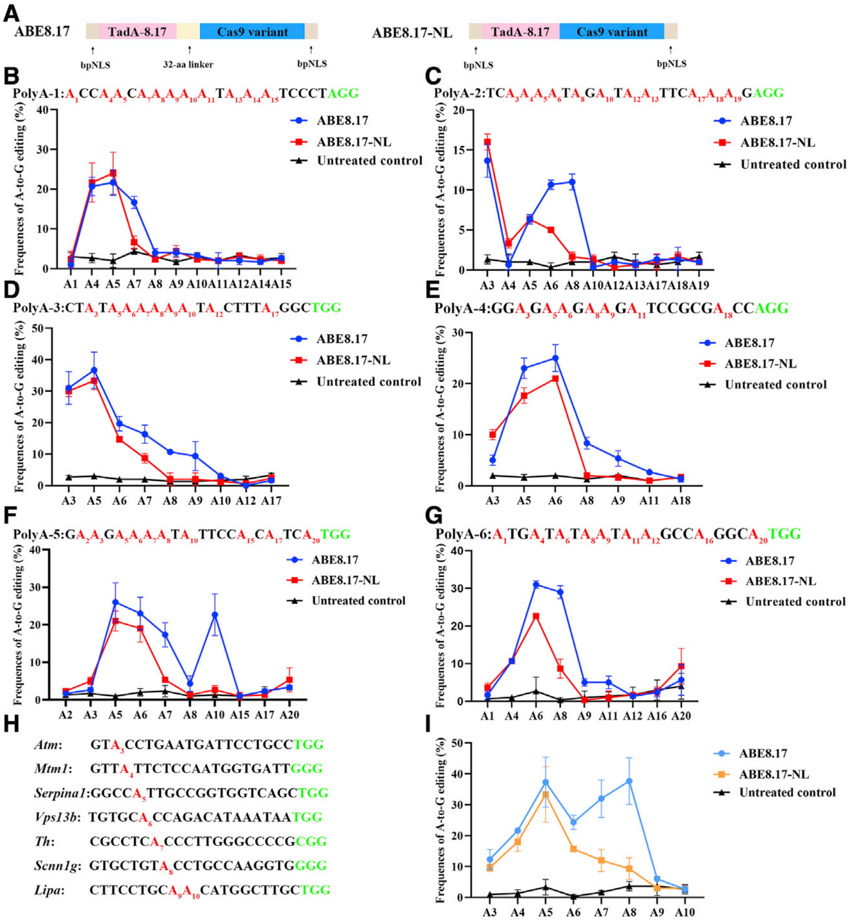

Figure 4. Efficient base editing with a narrowed window using ABE8.17-NL

(A) The architecture of ABE8.17 and ABE8.17-NL. bpNLS, bipartite nuclear localization signal. (B–G) Base-editing efficiency at polyA sites in HEK293FT cells using ABE8.17 and ABE8.17-NL. Bars represent mean values, and error bars represent the SDs of three independent biological replicates. (H) Protospacers and PAM (green) sequences of the six target sites (containing A bases at different positions). Target A bases are indicated in red. Subscript numbers indicate the positions of the adenine bases relative to the PAM. (I) Base-editing efficiency and precision at target A bases using ABE8.17 and ABE8.17-NL. Bars represent mean values, and error bars represent the SDs of three independent biological replicates.

(2–4 nts) in human HEK293FT cells. However, compromised editing efficiencies at 4 out of 7 sites tested were also determined by using ABE8.17-NL. We attempted to improve efficiency by introducing the point mutations (N127K, Q154R) [24] inTadA-8.17, which showed superior editing activity to NG-ABEmax. Our results showed that the editing efficiency was improved by 4% at A8 and decreased 6% at A5 and A6 by introducing N127K and Q154R (data not shown). Thus, we hypothesize that there is a balance betweenthe editing efficiency and editing window size, as reported in previous studies.[24–26]

In summary, the ABE8.17 system can be used to efficiently induce base mutations at target sites that are difficult to edit using ABE7.10, and provide an efficient method for mimicking clinical disease mutations in rabbits.

MATERIALS AND METHODS

Ethics statement

New Zealand white rabbits, Lianshan black rabbits and ICR mice were obtained from the Laboratory Animal Center of Jilin University (Changchun, China). All animal studies were conducted according to experimental practices and standards approved by the Animal Welfare and Research Ethics Committee of Jilin University (IACUC number: pzpx20200102027).

Plasmid construction

The ABE8e, ABE8.17-m, ABE8.20-m, pCMV-ABE7.10, pCMV_ABEmax, and pCMV-T7-SpRY-P2A-EGFP plasmids were obtained from Addgene (#138489, #136298, #136300, #102919, #112095, and #139989, respectively). The construction of Nme2-ABEmax was described in detail in our previously published study.[13] The open reading frames of nNme2Cas9 and SpRYCas9 were amplified by PCR for subsequent assembly into a base-editing architecture backbone using a ClonExpress Ultra One Step Cloning Kit (Vazyme, Nanjing, China). TadA8.17 DNA fragments were synthesized and cloned into nNme2-ABE8.17 and SpRY-ABE8.17 by GenScript Biotech (Nanjing, China). The D10A mutation was introduced into the SpRY-ABE8.17 plasmid. Site-directed mutagenesis was performed using a Fast Site-Directed Mutagenesis Kit (TIANGEN, Beijing, China). To construct the ABE8.17-NL plasmid, the reading frame encoding TadA-8.17 was amplified by PCR and used to replace TadA-8.17 and the linker fragment within ABE8.17-m, thus generating ABE8.17-NL. The sgRNA plasmid was constructed and inserted into the PUC57 and 74,707 vectors. Spacer

bases are indicated in red. (E) Sanger sequencing chromatograms of DNA from WT and *Lmna* (p.L530P) rabbits (#5 and #6). The red arrow indicates the substituted nucleotide. Relevant codon identities at the target site are presented beneath the DNA sequence. (F) Characterization of the targeted modifications in *Lmna* (p.L530P) rabbits by deep sequencing. (G) Lamin A/C protein expression was determinated by western blotting. (H) The L530P mutation in exon 9 of the *Lmna* gene induces intron retention. Primer F spanned exon 9 (4 bp) and intron 9. The yellow box (Int) represents the portion of intron 9. (I) Expression of the *Lmna* gene was determined by quantitative real-time PCR with specific primers targeting the exon and intron. M, which shows the DL2000 ladder, indicates band size. (J) Sanger sequencing chromatograms of the quantitative real-time PCR product.

oligos and sgRNA scaffold oligos were synthesized and cloned into the 74,707 and pUC57-sgRNA expression vectors.

Cell culture and transfection

Human kidney epithelial cells (HEK293FT) were cultured in Dulbecco's modified Eagle's medium (DMEM) supplemented with 10% fetal bovine serum (HyClone), 2 mM GlutaMAX (Life Technologies), 100 U/mL penicillin, and 100 mg/mL streptomycin and incubated at 37°C in an atmosphere containing 5% CO_2. The cells were seeded into 6-well plates at a density of 120,000 cells per well and transfected using Hieff Trans Liposome nucleic acid transfection reagent (Yeasen Biotechnology, Shanghai, China). The sequences of the sgRNAs are listed in Table S3. The isolated DNA was amplified by PCR with the TIANamp Genomic DNA Kit (TIANGEN, Beijing, China) according to the manufacturer's instructions. The primer sequences are listed in Table S4.

Estimation of editing frequency

EditR (https://moriaritylab.shinyapps.io/editr_v10/) online software[27] and the TIDE web tool (https://tide.deskgen.com/)[28] were applied to estimate the editing frequency of Sanger sequencing.

mRNA and gRNA preparation

ABE8.17 and SpRY-ABE8.17 were linearized with NotI and transcribed *in vitro* using the HiScribe T7 ARCA mRNA Kit (NEB). The sgRNAs were then amplified and transcribed *in vitro* using the MAXIscript T7 Kit (Ambion). mRNA was purified using the RNeasy Mini Kit (Qiagen) according to the manufacturer's protocol.

Microinjection and embryo transfer

The protocol used for the microinjection of pronuclear-stage embryos was described in detail in our previously published study.[29] Briefly, ABE mRNA (100 ng/μL) and sgRNA (50 ng/μL) were coinjected into the cytoplasm of pronuclear-stage embryos. The injected embryos were cultured for 30–60 min, after which approximately 30–50 injected embryos were transferred into the oviduct of the recipient mother.

Single-embryo PCR amplification and rabbit genotyping

The injected embryos were collected at the blastocyst stage. Genomic DNA was extracted from each embryo using lysis buffer (1% N-P40) at 56°C for 60 min and 95°C for 10 min in a Bio-Rad PCR amplifier. The genomic region surrounding the target site was PCR amplified and subjected to Sanger sequencing.

Genomic DNA was extracted from the skin tissue collected by ear clipping in newborn rabbits. The genomic regions surrounding the target site were PCR amplified and then subjected to Sanger sequencing and deep sequencing. Targeted sites were amplified from genomic DNA using Phusion polymerase (Thermo Fisher Scientific). The paired-end sequencing of PCR amplicons was performed by Sangon Biotech (Shanghai) using an Illumina MiSeq instrument. Sequences for all the primers used for genotyping are listed in Table S4.

Real-time quantitative PCR and western blotting

Total RNA was isolated with TRIzol-A+ reagent (TIANGEN, Beijing, China) according to the manufacturer's instructions. cDNA was synthesized with DNase I (Fermentas)-treated total RNA using the BioRT cDNA First Stand Synthesis Kit (Bioer Technology, Hangzhou, China). Quantitative real-time PCR was performed using the BioEasy SYBR Green I Real-Time PCR Kit (Bioer Technology, Hangzhou, China) with the Bio-Rad Iq5 Multicolour Real-Time PCR Detection System. Relative gene expression normalized to GAPDH expression was determined by the $2^{-\Delta\Delta CT}$ method. All gene expression data were obtained at least three times.

For western blotting, the ear tissues of pup #6 and WT rabbits were homogenized in 150 μL lysis buffer. The protein concentrations were measured by the Braford method (Bio-Rad). An anti-Lamin A/C polyclonal antibody (1:2,000; Proteintech) and an anti-β-actin monoclonal antibody (1:2,000; Proteintech) were used in this experiment.

Off-target assay

Twenty-two potential off-target sites (POTs) of sgRNAs were predicted to analyse site-specific edits according to Cas-OFFinder (http://www.rgenome.net/cas-offinder/)[30] by performing five general steps: (1) select PAM Type according to specific CRISPR/Cas-derived RNA-guided endonucleases, including SpCas9 from *Streptococcus pyogenes*(PAM:NGG), SpCas9 from *Streptococcus pyogenes* (PAM:NRG,R=A or G), complementary SpCas9 from *Streptococcus pyogenes*(PAM:NCC), and SpCas9 from *Streptococcus pasteurianus* (PAM: NNGTGA); (2) select the vertebrate genomes and the corresponding *Oryctolagus cuniculus* (OryCun2) - rabbit genome; (3) write crRNA sequences without PAM sequences; (4) select the mismatch number; and (5) click submit. Targeted sites were amplified from genomic DNA using Q5 polymerase (NEB). Mutations were detected by deep sequencing via HiTOM analysis.[31] All primers employed for the off-target assay are listed in Data S1.

Statistical analyses

All data are expressed as the mean ± SD, with at least three individual determinations carried out in all experiments. The data were analyzed with a two-tailed unpaired t test (both populations having the same SD) or an unpaired *t*-test with Welch's correction (no assuming equal SDs) using GraphPad Prism software 8.0. $p < 0.05$ indicated statistical significance (*$p < 0.05$, **$p < 0.01$, ***$p < 0.001$).

Availability of data and materials

Deep sequencing data from this work have been deposited in the Sequence Read Archive under accession code PRJNA768986 (https://www.ncbi.nlm.nih.gov/sra/PRJNA768986).

SUPPLEMENTAL INFORMATION

Supplemental information can be found online at https://doi.org/10.1016/j.omtn.2022.01.019.

ACKNOWLEDGMENTS

This study was financially supported by the National Key Research and Development Program of China Stem Cell and Translational Research (2017YFA0105101) and The National Natural Science Foundation of China (Nos.32170543 and 31970574). The authors thank Peiran Hu and Nannan Li for assistance at the Embryo Engineering Center for critical technical assistance.

AUTHOR CONTRIBUTIONS

D.Z., Z.L. and L.L. conceived and designed the experiments. D.Z.,Y.Q. and J.L. performed the experiments. D.Z. and Z.L. analyzed the data. J.L. and Y.Q. contributed reagents/materials/analysis tools. D.Z., J.L. and Z.L. wrote the paper. All authors read and approved the final manuscript.

DECLARATION OF INTERESTS

The authors declare no conflict of interests.

REFERENCES

1. Gaudelli, N.M., Komor, A.C., Rees, H.A., Packer, M.S., Badran, A.H., Bryson, D.I., and Liu, D.R. (2017). Programmable base editing of A●T to G●C in genomic DNA without DNA cleavage. Nature *551*, 464–471.
2. Hua, K., Tao, X., Yuan, F., Wang, D., and Zhu, J.K. (2018). Precise A·T to G·C base editing in the rice genome. Mol. Plant *11*, 627–630.
3. Yan, F., Kuang, Y., Ren, B., Wang, J., Zhang, D., Lin, H., Yang, B., Zhou, X., and Zhou, H. (2018). Highly efficient A·T to G·C base editing by Cas9n-guided tRNA adenosine deaminase in rice. Mol. Plant *11*, 631–634.
4. Ryu, S.M., Koo, T., Kim, K., Lim, K., Baek, G., Kim, S.T., Kim, H.S., Kim, D.E., Lee, H., Chung, E., et al. (2018). Adenine base editing in mouse embryos and an adult mouse model of Duchenne muscular dystrophy. Nat. Biotechnol. *36*, 536–539.
5. Zhang, W., Aida, T., Del Rosario, R.C.H., Wilde, J.J., Ding, C., Zhang, X., Baloch, Z., Huang, Y., Tang, Y., Li, D., et al. (2020). Multiplex precise base editing in cynomolgus monkeys. Nat. Commun. *11*, 2325.
6. Qin, W., Lu, X., Liu, Y., Bai, H., Li, S., and Lin, S. (2018). Precise A●T to G●C base editing in the zebrafish genome. BMC Biol. *16*, 139.
7. Liu, Z., Lu, Z., Yang, G., Huang, S., Li, G., Feng, S., Liu, Y., Li, J., Yu, W., Zhang, Y., et al. (2018). Efficient generation of mouse models of human diseases via ABE- and BE-mediated base editing. Nat. Commun. *9*, 2338.
8. Liu, Z., Chen, M., Chen, S., Deng, J., Song, Y., Lai, L., and Li, Z. (2018). Highly efficient RNA-guided base editing in rabbit. Nat. Commun. *9*, 2717.
9. Richter, M.F., Zhao, K.T., Eton, E., Lapinaite, A., Newby, G.A., Thuronyi, B.W., Wilson, C., Koblan, L.W., Zeng, J., Bauer, D.E., et al. (2020). Phage-assisted evolution of an adenine base editor with improved Cas domain compatibility and activity. Nat. Biotechnol. *38*, 883–891.
10. Gaudelli, N.M., Lam, D.K., Rees, H.A., Solá-Esteves, N.M., Barrera, L.A., Born, D.A., Edwards, A., Gehrke, J.M., Lee, S.J., Liquori, A.J., et al. (2020). Directed evolution of adenine base editors with increased activity and therapeutic application. Nat. Biotechnol. *38*, 892–900.
11. Koblan, L.W., Doman, J.L., Wilson, C., Levy, J.M., Tay, T., Newby, G.A., Maianti, J.P., Raguram, A., and Liu, D.R. (2018). Improving cytidine and adenine base editors by expression optimization and ancestral reconstruction. Nat. Biotechnol. *36*, 843–846.
12. Walton, R.T., Christie, K.A., Whittaker, M.N., and Kleinstiver, B.P. (2020). Unconstrained genome targeting with near-PAMless engineered CRISPR-Cas9 variants. Science *368*, 290–296.
13. Liu, Z., Chen, S., Jia, Y., Shan, H., Chen, M., Song, Y., Lai, L., and Li, Z. (2021). Efficient and high-fidelity base editor with expanded PAM compatibility for cytidine dinucleotide. Sci. China Life Sci. *64*, 1355–1367.
14. Wang, Y., Fan, N., Song, J., Zhong, J., Guo, X., Tian, W., Zhang, Q., Cui, F., Li, L., Newsome, P.N., et al. (2014). Generation of knockout rabbits using transcription activator-like effector nucleases. Cell Regen. *3*, 3.
15. Oetting, W.S., and King, R.A. (1999). Molecular basis of albinism: mutations and polymorphisms of pigmentation genes associated with albinism. Hum. Mutat. *13*, 99–115.
16. Bonne, G., Di Barletta, M.R., Varnous, S., Bécane, H.M., Hammouda, E.H., Merlini, L., Muntoni, F., Greenberg, C.R., Gary, F., Urtizberea, J.A., et al. (1999). Mutations in the gene encoding lamin A/C cause autosomal dominant Emery-Dreifuss muscular dystrophy. Nat. Genet. *21*, 285–288.
17. Spritz, R.A., Oh, J., Fukai, K., Holmes, S.A., Ho, L., Chitayat, D., France, T.D., Musarella, M.A., Orlow, S.J., Schnur, R.E., et al. (1997). Novel mutations of the tyrosinase (TYR) gene in type I oculocutaneous albinism (OCA1). Hum. Mutat. *10*, 171–174.
18. Oetting, W.S. (2000). The tyrosinase gene and oculocutaneous albinism type 1 (OCA1): a model for understanding the molecular biology of melanin formation. Pigment Cell Res. *13*, 320–325.
19. Zhou, C., Sun, Y., Yan, R., Liu, Y., Zuo, E., Gu, C., Han, L., Wei, Y., Hu, X., Zeng, R., et al. (2019). Off-target RNA mutation induced by DNA base editing and its elimination by mutagenesis. Nature *571*, 275–278.
20. Zuo, E., Sun, Y., Wei, W., Yuan, T., Ying, W., Sun, H., Yuan, L., Steinmetz, L.M., Li, Y., and Yang, H. (2019). Cytosine base editor generates substantial off-target single-nucleotide variants in mouse embryos. Science *364*, 289–292.
21. Sakurai, T., Shindo, T., and Sato, M. (2017). Noninheritable Maternal Factors Useful for Genetic Manipulation in Mammals. Results Probl. Cell Differ. *63*, 495–510.
22. Yuan, L., Sui, T., Chen, M., Deng, J., Huang, Y., Zeng, J., Lv, Q., Song, Y., Li, Z., and Lai, L. (2016). CRISPR/Cas9-mediated GJA8 knockout in rabbits recapitulates human congenital cataracts. Sci. Rep. *6*, 22024.
23. Sui, T., Yuan, L., Liu, H., Chen, M., Deng, J., Wang, Y., Li, Z., and Lai, L. (2016). CRISPR/Cas9-mediated mutation of PHEX in rabbit recapitulates human X-linked hypophosphatemia (XLH). Hum. Mol. Genet. *25*, 2661–2671.
24. Fu, J., Li, Q., Liu, X., Tu, T., Lv, X., Yin, X., Lv, J., Song, Z., Qu, J., Zhang, J., et al. (2021). Human cell based directed evolution of adenine base editors with improved efficiency. Nat. Commun. *12*, 5897.
25. Yan, D., Ren, B., Liu, L., Yan, F., Li, S., Wang, G., Sun, W., Zhou, X., and Zhou, H. (2021). High-efficiency and multiplex adenine base editing in plants using new TadA variants. Mol. Plant *14*, 722–731.
26. Komor, A.C., Kim, Y.B., Packer, M.S., Zuris, J.A., and Liu, D.R. (2016). Programmable editing of a target base in genomic DNA without double-stranded DNA cleavage. Nature *533*, 420–424.
27. Kluesner, M.G., Nedveck, D.A., Lahr, W.S., Garbe, J.R., Abrahante, J.E., Webber, B.R., and Moriarity, B.S. (2018). EditR: A Method to Quantify Base Editing from Sanger Sequencing. Crispr. J. *1*, 239–250.
28. Brinkman, E.K., Chen, T., Amendola, M., and Van Steensel, B. (2014). Easy quantitative assessment of genome editing by sequence trace decomposition. Nucleic Acids Res. *42*, e168.
29. Song, Y., Yuan, L., Wang, Y., Chen, M., Deng, J., Lv, Q., Sui, T., Li, Z., and Lai, L. (2016). Efficient dual sgRNA-directed large gene deletion in rabbit with CRISPR/Cas9 system. Cell Mol Life Sci *73*, 2959–2968.
30. Bae, S., Park, J., and Kim, J.S. (2014). Cas-OFFinder: a fast and versatile algorithm that searches for potential off-target sites of Cas9 RNA-guided endonucleases. Bioinformatics *30*, 1473–1475.
31. Liu, Q., Wang, C., Jiao, X., Zhang, H., Song, L., Li, Y., Gao, C., and Wang, K. (2019). Hi-TOM: a platform for high-throughput tracking of mutations induced by CRISPR/Cas systems. Sci China Life Sci *62*, 1–7.

OMTN, Volume *27*

Supplemental information

Highly efficient A-to-G base editing by ABE8.17 in rabbits

Ding Zhao, Yuqiang Qian, Jinze Li, Zhanjun Li, and Liangxue Lai

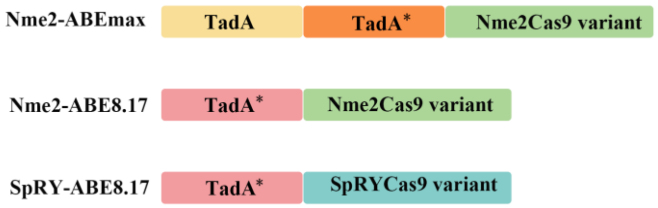

Supplementary Figure 1

The architecture of Nme2-ABEmax, Nme2-ABE8.17 and SpRY-ABE8.17.

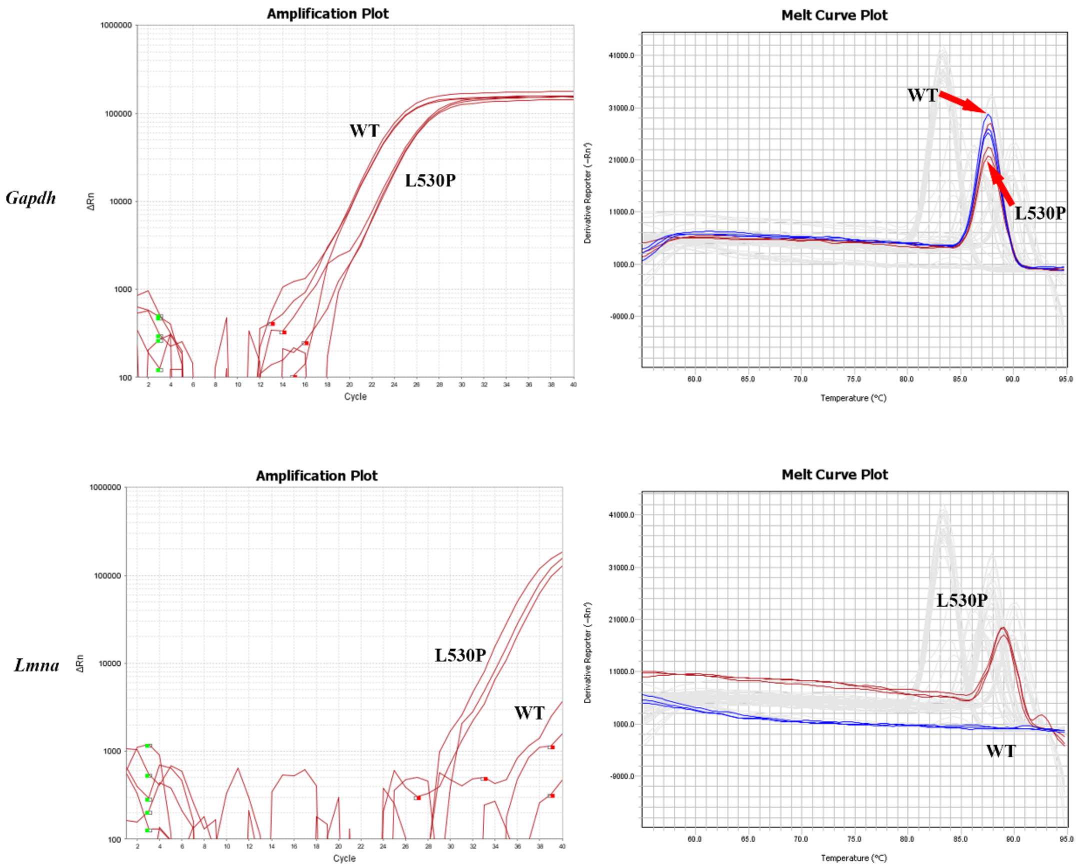

Supplementary Figure 2

Expression of the *Lmna* gene was determined by qRT–PCR with specific primers targeting the exon and intron.

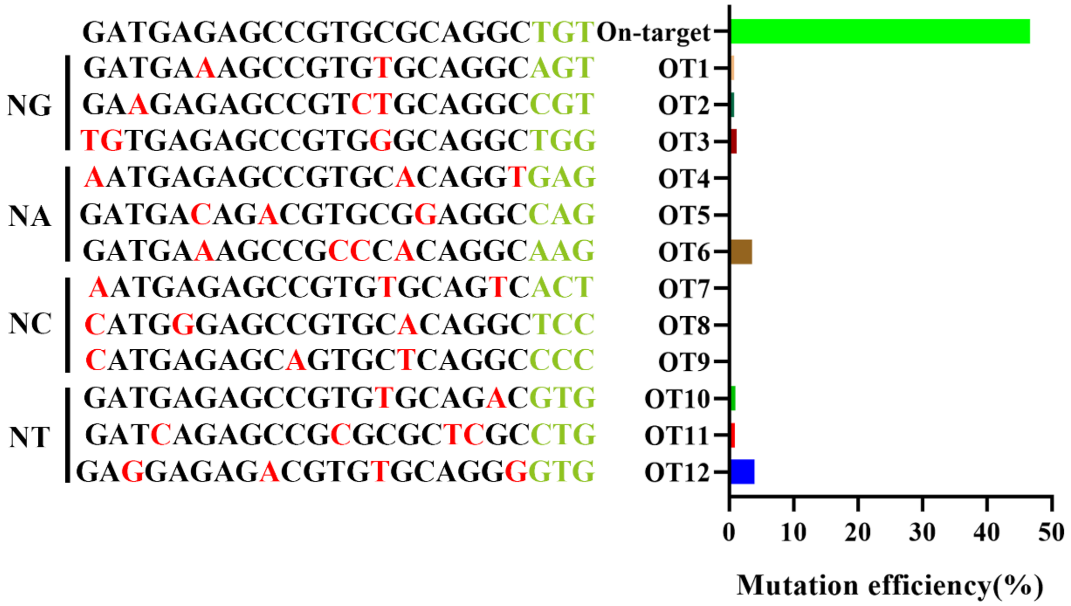

Supplementary Figure 3

Analysis of off-target effects in *Lmna* mutant rabbits by deep sequencing of HiTOM analysis. The potential off-target sites (POTs) in the rabbit genome for sgRNA were predicted to analyze site-specific edits with Cas-OFFinder (http://www.rgenome.net/cas-offinder/). Deep sequencing was carried out to analyse the PCR products of the POTs. All the primer sequences for the off-target assay are listed in Data S1.

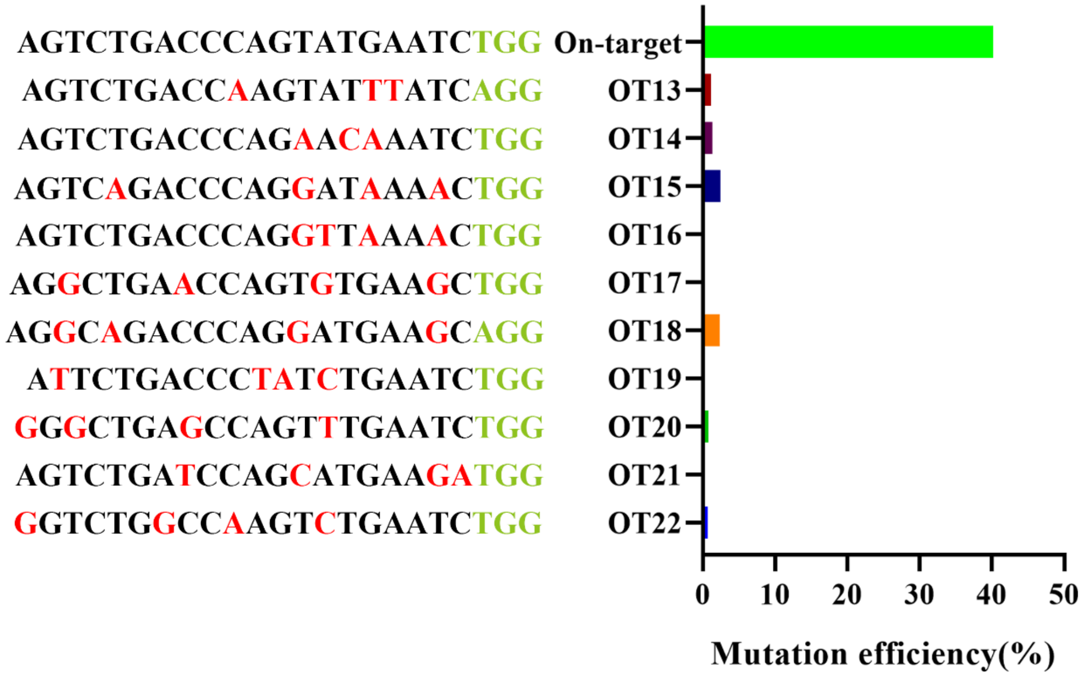

Supplementary Figure 4

Analysis of off-target effects in *Tyr* mutant rabbits by deep sequencing of HiTOM analysis. The potential off-target sites (POTs) in the rabbit genome for sgRNA were predicted to analyze site-specific edits with Cas-OFFinder (http://www.rgenome.net/cas-offinder/). Deep sequencing was carried out to analyze the PCR products of the POTs. All the primer sequences for the off-target assay are listed in Data S1.

Table S1 Summary of mouse embryo base editing rate using ABE8.17 system

Target gene	Base editor	No. of blastocysts	Mutant ratio (%)	
			No. of mutants	No. of target mutants
Tyr	ABE7.10	7	0(0)	0(0)
	ABE8.17	13	12(92)	12(92)
Dmd	ABE7.10	9	0(0)	0(0)
	ABE8.17	11	4(36)	3(27)
Lmna	ABE7.10	11	5(45)	0(0)
	ABE8.17	9	9(100)	3(33)

Table S2 Summary of rabbit embryo base editing rate using ABE8.17 system

Target gene	Base editor	No. of blastocysts	Mutant ratio (%)	
			No. of mutants	No. of target mutants
Tyr-1	ABE7.10	6	0(0)	0(0)
	ABE8.17	5	4(80)	4(80)
Tyr-2	ABE7.10	7	0(0)	0(0)
	ABE8.17	9	3(33)	3(33)
Lmna	ABE7.10	9	3(33)	0(0)
	ABE8.17	11	9(82)	4(36)
	SpRY-ABE8.17	9	5(56)	5(56)

Table S3. Target sites in human cells used in this study. Target nucleotides (red)

**Target site**	sequence (5'-3')	PAM
**ABE-site1**	GAAGA$_5$CCA$_8$AGGATAGACTGC	GGG
**ABE-site2**	GAACA$_5$CA$_7$AAGCATAGACTGC	GGG
**ABE-site3**	GATGA$_5$GA$_7$TA$_9$ATGATGAGTCA	AGG
**ABE-site4**	GATGA$_5$GA$_7$A$_8$GGAGAAGTTCTT	TGG
**ABE-site5**	GAATA$_5$CTA$_8$AGCATAGACTCC	AGG
**ABE-site6**	GTGTA$_5$A$_6$GACCTCAAAAGCAC	AGG
**ABE-site7**	AAATA$_5$A$_6$TGCCATCTTCCGCT	AGG
**NAA**	GGCTGCACAACCAGTGGAGG	CAA
**NAC**	GGGTCAGACGTCCAAAACCA	GAC
**NAT**	GTCCACTTCCCCTCCCACTA	CAT
**NAG**	GCAGGAGGTGGGGAAGGCCG	AAG
**NGA**	GAGGAGGAAGGGCCTGAGTC	CGA
**NGC**	GACA$_4$GGCA$_8$GGGGCACCGCGG	CGC
**NGG**	AAATA$_5$A$_6$TGCCATCTTCCGCT	AGG
**NGT**	GTTCCAGAACCGGAGGACAA	AGT
**Nm1**	ATCTGTCCCCTCCACCCCACAGT	GGGGCC
**Nm2**	ATGTTCCAGCTTCCTGGGTCTGC	AGGTCC

**Table S4.** Primers used for genotyping in this study	
**Target site**	**Sequence (5'-3')**
**ABE-site1**	F GAGGCATGAGGATCACTTGAG
	R TCAGCTCCAACAGCACTTT
**ABE-site2**	F ACTGCCATTCTACCAACAATAGA
	R AAGCAGGTGATTACAGGATTGA
**ABE-site3**	F ACAGTGGGTTAATAGGAGAATGAG
	R TCCCAAAGTGCTGGGATTT
**ABE-site4**	F CACCCCTTCAGTCCATGCTT
	R TCTGATGGGGAGGAACGAGT
**ABE-site5**	F AGAAGGTAAGTGCATGGTAAGG
	R CCAGATTTCTCAGCCTCTTTCT
**ABE-site6**	F GGAGAACCATAGGCAGAAGAAT
	R CTACTGAGTTGCTTGAGCTCTTA
**ABE-site7**	F GCGAGGGTTACAGTTCTGTTTAG
	R GCCTCCTGCTGTCATTGATATT
**NAA**	F CATTTCGCGGATGTTCCAATC
	R CCTGGAGATTTGGGTTCTCTC
**NAC**	F CTCTGGTGCTGTGTGACTA
	R GGACGAGGGAAATTTGAAATCG
**NAT**	F AAGCGATGAGCAAGCTACC
	R AGAACCTCATTCAGTGGTTCATTA
**NAG**	F CGTTACCACCTGGTGCAG
	R CCCAGAAGCCAGTGGAC
**NGA**	F TAGCCTCAGTCTTCCCATCA
	R GTTGCCCACCCTAGTCATT
**NGC**	F GACAGACCACAAGCAGGTAAA
	R TTTCAACCCGAACGGAGAC
**NGG**	F GCGAGGGTTACAGTTCTGTTTAG
	R GCCTCCTGCTGTCATTGATATT
**NGT**	F GGGGCCTCCTGAGTTTCTCATCTG
	R GGTTGCCCACCCTAGTCATTGGAG
**Nm1**	F CAGCTAGTCTTCTTCCTCCAAC
	R CCAGATAAGGAATCTGCCTAACA
**Nm2**	F TCTTCTCCTGTGGTGGAGT
	R CTGTGCCTCAACCTCCTAAC
Tyr	F TGCCAGAAAGCTGAATGATACT
	R CTTCGCTTCCCTTTCCCTTT
Dmd	F TGGAGTTATATCCCTGTGGAAAC
	R CCAAGATAGGCCTCATGACAA
***Lmna* (mouse)**	F CCACCGAAGTTCACCCTAAA
	R GGCTGACTTCCACCCATTAT
Tyr-1	F GTACAATAGCCGACAGAGCTTAT

	R CTGCCTGGTGTTTCTGTTTATTT
Tyr-2	F CTCCCAGTGATGAGAAACCTAAC
	R CTCCCAGTGATGAGAAACCTAAC
***Lmna* (rabbit)**	F GACAGTAGCACGGAGTGAG
	R GCTAAGGAACTTGCTCCCA
**PolyA-1**	F CCCAATTCCACGATGAACAGA
	R CTCCAAAGCTGAAGGCAAGA
**PolyA-2**	F AGTTGTTACCGTTGGGACTATTT
	R AAGATCGCGCCATTGTACTC
**PolyA-3**	F GGGCATTTGGAAGTCAGTAGTA
	R TTTGGGAGGTGATCAGGTTATG
**PolyA-4**	F CGACAACACTTGTTATGGAAAGG
	R AGACTGGGAAAGGCAATGG
**PolyA-5**	F GTATGTGAGTGACCACCAAGAG
	R CTCCCTCTTATCCATGTGTTAGC
**PolyA-6**	F ACCTGTAATCCTAGCACTTTGG
	R AGCAAGGAGCAACAGAAGAA
Atm	F TTTGTATGGCTGTGGTGGAG
	R TTTCCTGTGTCTCCCTGAATTT
Mtm1	F GGATTATTGATTTGGCACCCTTATT
	R TCTCCAGTCTGGTATGGTACTT
Serpina1	F AGCTCCTTGACCAAATCCAC
	R CCAGTCCAACAGCACCAATA
Vps13b	F GCTTTCTGCCATAATGAAGTCTG
	R AAAGAGGGCTGAACACAATCTA
Th	F CTTGTCCAGCTCTGACACTT
	R TCTCCTTCTCTCTCTCTTCCTG
Scnn1g	F CCTCACTGTGGCTGGTTTAT
	R GGGAGAATCTAGGCTGCTTTC
Lipa	F ACAACTTCAGAGTTACCACCTATC
	R TACCTTGCCAGTGCTGTTT

Table S5. Generation of *Lmna* and *Tyr* rabbits using ABE8.17 systems.

Target site	Embryos transferred	No. of offspring	No. of mutants	No. of target mutants	Frequencies of target mutations
Lmna (p.L530P)	42	7	2(28.57%)	1(14.29%)	45.41%
Tyr (p.T325A)	46	5	3(60.00%)	3(60.00%)	(40.03±26.82%)

Data are presented as mean ± SD.
